# Supplementary material for: Are carbon emissions trading and green financial instruments synergistic? -Comprehensive quantitative research based on content analysis
Source: PLoS One. 2024 Mar 7;19(3):e0298601. doi: 10.1371/journal.pone.0298601 (PMC10919601; doi:10.1371/journal.pone.0298601)
Supplement: S1 Data — (ZIP) [file pone.0298601.s001.zip › Data/地级市Z_F工作报告.zip!/╔╜╢1⁄2/║╩╘≤╩╨/└┤╫╘╣1⁄2╓┌║┼í╛┬φ┐╦╩2╛▌═°í┐_2018.docx]

各位代表：

　　现在，我代表菏泽市人民政府向大会报告工作，请予审议。请市政协各位委员、各位列席的同志提出意见。

　　一、2017年工作回顾

　　过去的一年，面对发展任务重、标准高、挑战多、压力大等多重集中考验，在市委的坚强领导下，我们认真贯彻中央和省委、省政府的决策部署，迎难而上，聚力攻坚，全力扩大优质增量，大力推动动能转换，始终坚持补短板、守底线、防风险、惠民生，经济社会发展稳中有进、持续向好。预计全年实现地区生产总值2880亿元，增长8.6%左右；地方一般公共预算收入186.5亿元，增长6.1%；城乡居民人均可支配收入24110元、11720元，分别增长9%、9.5%。主要经济指标增幅位居全省前列，经济结构持续优化，生态环境显著改善，社会保持和谐稳定，人民群众的获得感、幸福感明显增强。

　　一是着力提高推动发展的能力，履职服务水平不断提升。面对新任务、新考验、新机遇，主动适应，积极作为，不断以新理念、新方式、新措施推动新形势下的各项工作。以创新的思路谋划工作。牢牢把握国家发展大势和政策趋向，以前瞻性理念、创造性思维精心谋划实施电子商务、返乡创业、政府和社会资本合作、棚户区改造、为村建设等工作，实现了领先一步发展。坚持培训先行，开展各类培训570余场次，提高了各级领导干部适应形势、抢抓机遇、科学决策、推动发展的能力和水平。以更有效的举措推动落实。细化分解248项政府重点工作，明确责任领导、责任部门、责任人，完成情况定期向社会公开，主动接受人民群众的监督。把企业对地方财政收入贡献、为村建设、新增常住人口等纳入重点经济工作考核，带动全局工作。坚持在一线发现问题、解决问题，健全工作调度推进机制，增强了工作的精准性。注重总结推广典型，并将经验固化为制度和机制，推动工作开展。以更大的力度优化服务。深化“放管服”改革，取消、下放行政审批事项10项，取消中介服务收费项目17项，加快推行“多证合一、一照一码” “容缺审批”“只跑一趟”等服务方式，激发了创新创业活力，全市新增市场主体13.98万户。创新行政管理体制，分别下放市经济开发区、菏泽高新区行政执法权力158项、210项。以更高的要求依法行政。积极推进法治政府建设，切实完善重大事项决策程序，实行了行政执法“双随机一公开”制度。自觉接受人大工作监督、法律监督和政协民主监督，认真落实市人大常委会审议意见，办结市人大代表建议124件、市政协提案268件。规范预算管理，市级“三公经费”预算全部公开，同比下降32.6%。严格落实中央八项规定精神，持续加大“四风”问题监督检查力度，政府运行效率进一步提高。

　　二是强力推进新旧动能转换，经济新引擎加速成长。坚持以新旧动能转换统领经济工作，突出“四新”促“四化”，一批引领支撑未来发展的新引擎不断涌现、加速成长。产业升级步伐加快。积极谋划实施新旧动能转换重大项目，进入省项目库数量位居全省前列，菏泽新旧动能转换实施规划编制工作基本完成。着力推进工业强市战略，深入实施百强企业培育计划，预计全市规模以上工业增加值增长10%左右。纳税过亿元的工业企业达到15家，新增3家。东明石化牵头成功组建山东炼化能源有限公司。预计全市完成技改投资400亿元、增长12%，高新技术产业产值占规模以上工业总产值的比重达到33.7%、同比提高1个百分点。新增挂牌上市企业22家。加快发展现代农业，新增市级以上农民合作社示范社37家、示范家庭农场14家、省级以上标准化示范养殖场35个、省级农业标准化生产基地15个、“三品一标”产品123个、市级以上“一村一品”示范村镇32个。大力发展现代服务业，济铁、传化等现代物流园区建成投用，天华新媒体广告产业园被认定为“国家广告产业园区”“全国广告业创新创业示范基地”。预计全市服务业增加值占地区生产总值的比重达到38.2%、同比提高0.8个百分点，其中现代服务业增加值占服务业增加值的51.9%、同比提高0.7个百分点。外向型经济取得新进展，预计全市完成进出口总额390亿元、增长12%，出口商品自贸协定利用率65.4%、全省最高。互联网经济领先优势持续扩大。淘宝村、淘宝镇分别由去年的61个、6个发展到168个、24个，分别占全国的1/12、1/10，均占全省的2/3，数量在全国地级市中均居第一位。实现了县区淘宝村全覆盖，曹县大集镇32个村全部成为淘宝村。建成电商园区48个，入驻企业2594家，产品上行率由1.3：1提高到1.52：1，预计全市完成电商交易额2050亿元、增长70%。大力发展新实体经济，规模以上企业触网率达到51.5%，“1688菏泽产业带”整体运行全省第一。国家电子商务示范城市创建扎实推进，第五届中国淘宝村高峰论坛在我市成功举办，打造了高端化、特色化、国际化电商盛会。新人口红利加速释放。大力发展“归雁经济”。出台支持政策20条，在全国率先设立市级返乡创业管理服务中心，新增返乡创业服务站12个、达到34个，各县区设立的服务站达到了73个。组织开展了全方位、多层次的宣传推介活动，3.1万人返乡创业，领办、创办经济实体1万余家，带动10万人就地就近就业。投融资改革取得新成效。科学策划筛选、积极储备推介政府和社会资本合作项目，通过财政部综合信息平台审核的项目249个，其中国家级示范项目8个，居全国地级市首位。争取上级各种扶持资金374.2亿元。组建重组的机场投资、铁路投资、投资发展、财金、城投、金土地等6家公司，经营带动能力不断提升。

　　三是全力打好“五场攻坚战”，发展的可持续性显著增强。坚持保底线、守红线，组织实施了生态环保、脱贫攻坚、金融风险防控、社会稳定、安全生产五场攻坚战。以壮士断腕的勇气开展环保综合整治攻坚。以环保攻坚助推产业转型升级，取缔淘汰燃煤小锅炉7993台、取缔整改“散乱污”企业4313家，搬迁关闭禁养区养殖场1597家，年可减少用煤215万吨、废水排放103万吨。完成“双替代” 10.35万户，超额完成目标任务，年可节约燃煤2.75万吨。蓝天白云天数同比增加29天、达到253天，PM2.5、PM10年平均浓度分别下降13.58%、7.91%，重污染天数同比减少22天。全面实行“河长制”，市区新建污水管网31公里，三个污水处理厂完成扩容，新增日污水处理能力16万吨，三条主要河流出境断面水质实现达标。全力解决群众身边的环保问题1.7万个，立案查处环境违法案件1582件，群众对环保工作的满意度显著提升。以最大的政治责任实施精准扶贫精准脱贫。加大资金筹集投入力度，争取中央和省专项资金12.5亿元、同比增长308%。整合各类涉农资金34亿元用于脱贫攻坚。成立了全省首家省扶贫开发基金会市级办事处，募集资金1.04亿元。大病医疗商业补充保险在全省率先实现全覆盖。扎实推进黄河滩区脱贫迁建工程，18个村台开工建设，6个外迁社区全面启动。建成扶贫综合开发示范基地67个，实施产业扶贫项目2525个，建成扶贫车间3496个。脱贫40万人。扶贫工作经验在全国推广。以积极主动的作为防范化解系统性金融风险。压实了金融风险防控属地管理责任，实行金融风险定期排查交办制度，建立完善债委会工作机制，妥善处置化解了相关企业债务风险。积极开展打击逃废银行债务专项行动，抓好企业资金链风险化解，大力实施破圈断链工程，维护了区域金融安全。以强有力的责任担当维护社会稳定。认真落实退役士兵安置和权益保障政策。加强和改善社区治理，深入推进 “天网工程”建设，全市城乡监控探头保有量30余万个，覆盖率达到100%，被确定为全国公共视频监控建设联网应用示范城市。积极排查化解矛盾纠纷，确保了社会和谐稳定。以生命至上的理念推动安全生产。扎实开展安全生产大检查、危险化学品安全综合治理、“大快严”、百日攻坚治理、道路交通安全集中整治等专项行动，深入推进化工产业安全生产转型升级，全市生产安全事故起数、死亡人数继续保持“双下降”态势，安全生产形势持续稳定。

　　四是深入实施基础突破工程，城镇承载力辐射力不断提升。全面启动区域中心城市建设，中心城区首位度不断提升。重大基础设施工程顺利推进。菏泽机场全面开工，鲁南高铁、京九高铁菏泽段加快推进，德上高速、枣菏高速菏泽段集中施工，新万福河航道建设进展顺利，洙水河航道全面建成投用。城市路网不断完善。丹阳立交桥建成通车，东方红大街、中华路西段等11条道路升级改造全面完成，人民路、黄河路等6条道路升级改造有序推进，东方红西街、泰东路等13条断头路已经打通，人民路和中华路口、中山路和华英路口等11处路口渠化已经完工，跨赵王河、万福河8座桥梁即将完工，市区“二环、四横、六纵”道路网络基本形成。棚户区改造实现新的突破。实施重点棚改项目122个，完成征收18.2万户、2880万平方米，开工新建及货币化安置22.2万套，棚改总量居全国地级市首位。全市预售商品房面积784万平方米、增长30.4%。城镇基础配套更加完善。积极推进省级文明城市创建。拆除违规违法建筑667.7万平方米。市区新增集中供热小区57个、290万平方米，基本实现集中供热全覆盖。18个乡镇入选全国重点镇、占全省1/10。预计实现“三个市民化”18.5万人，全市常住人口城镇化率达到49%、提高1.64个百分点。

　　五是切实强化公共服务，群众获得感幸福感明显增强。始终着眼满足人民群众日益增长的美好生活需要，不断增加公共服务有效供给，市财政用于民生支出占比达到80.4%，同比提高0.6个百分点。逐步补齐民生短板。深入推进义务教育均衡发展、“全面改薄”、解决“大班额”等教育重点工作，全年投入66亿元，新建改造城区学校78所、农村幼儿园200所、农村校舍163.7万平方米、乡村教师周转宿舍18.6万平方米，招录教师4200人，县区全部通过国家义务教育均衡发展验收。职业教育加快发展，两个县区成为省级示范单位。公立医院法人治理结构改革顺利实施，医联体建设稳步推进，基本公共卫生服务走在全省前列。基层综合性文化服务中心覆盖率达到87.4%。积极推进城乡供水一体化，成为全省第二个以县为单位整建制城乡集中供水的市。完成农村改厕35万户。社会保障更加有力。城镇新增就业7.23万人，登记失业率控制在3.8%以内。实施贫困户危房改造12661户，城市低保月保障标准提高到420元，农村低保年保障标准提高到3600元、达到省定贫困线以上，最低月工资标准由1390元提高到1470元。社会治理更加高效。平安菏泽扎实推进，一村一法律顾问深入实施。推进市县乡村四级综治中心建设，乡镇一级平台已全部建成。新改扩建农村社区服务中心120处，城市社区服务中心实现全覆盖。集中开展“守护舌尖安全”综合治理，食品安全保障水平持续提升。

　　六是倾力办好民生实事，让群众共享更多发展成果。年初承诺的10个方面24项民生实事，除1项因政策调整暂缓实施外，23项全部完成。青年湖公园提升改造、环堤公园、体育公园完成规划设计。天香公园、火车站广场等28个市区公共区域实现无线网络免费开放。建成城市绿道23公里。市区新建垃圾中转站10个、停车场20处、公厕30个，新增新能源公交车100辆、公交线路5条，新增公共自行车1000辆、站点50座。实施了65岁以上老人免费健康查体和居民健康档案复核升级行动。建成“明厨亮灶”餐饮单位1247家、食品检测平台174个。建成“为村”平台5151个，占全国总数的87.9%。新建文化广场1000处，免费送戏下乡3000场。完成了811个小城镇、中心村、省定贫困村电网改造，7.2万眼机井全部通电，新改建农村公路4661.5公里。

　　妇女儿童、关心下一代、残疾人、老龄、慈善等事业加快发展，民族宗教、外事侨务、人民武装、防震减灾、邮政通讯、统计、物价、气象、人防、对台、史志、档案等工作都取得新成绩，圆满完成了市十九届人大一次会议确定的任务目标。在此，我谨代表市人民政府，向全市人民，向各级人大代表、政协委员，向各民主党派、工商联、人民团体和社会各界人士，向离退休老领导、老同志，向驻菏部队、武警官兵、公安干警，向所有关心支持菏泽建设和发展的同志们、朋友们，表示衷心的感谢并致以崇高的敬意!

　　我们也清醒地认识到，全市经济社会发展中还存在不少困难和问题。底子薄、欠账多的客观实际，使得发展中不平衡、不充分的矛盾在我市表现更为突出。实体经济遇到新的困难，有效投资支撑发展的能力不足，招商引资力度亟需加大。产业结构、产品结构需要进一步提升，科技创新能力不足，“四新”经济总体规模仍然偏小，新旧动能转换任重道远。坚守生态环保、安全生产、金融风险等发展底线的任务压力巨大。中心城区承载带动能力不足，城镇基础设施配套不完善，城乡融合水平不高。基本公共服务供给仍然不足，环境、交通、教育、医疗等民生领域短板明显。基层社会治理有不少差距。政府工作人员的专业素养、法制思维需要进一步提升，组织推动经济社会新发展的能力还需要进一步提高。对存在的这些困难和问题，我们一定高度重视，采取更加有力的措施认真加以解决。

　　二、今年工作的主要任务

　　当前，我市正处于蓄势待发、羽化蝶变，加快发展、后来居上的特殊历史阶段。认真分析新的形势，我们面临的发展机遇前所未有。习近平总书记亲临菏泽视察，对我市寄予后来居上的殷切期望，成为持续激发全市上下干事创业、担当作为、奋力赶超的不竭动力。党的十九大胜利召开，为我们指明了前进方向。新时代，我国社会主要矛盾发生新的变化，这一重大政治论断将对我市产生深远影响，有力促进我们更平衡更充分的发展。我国经济发展进入新时代，由高速增长阶段转向高质量发展阶段，为我们推动高质量发展、建设现代化经济体系创造了巨大空间。实施区域协调发展战略，进一步加大力度支持贫困地区加快发展，我市将享受更多政策支持。党的十九大把脱贫攻坚作为“三大攻坚战”之一，实施精准扶贫、精准脱贫，我市作为全省脱贫攻坚主战场，将汇集更多扶贫资源。机场、高铁、高速公路等重大基础设施密集实施，交通区位优势、后发崛起优势进一步凸显。国家持续支持棚户区改造，为我们改善人居环境、提升城市形象、完善城市功能创造了有利条件。电子商务领先发展，返乡创业蔚然成风，政府和社会资本合作项目走在全省前列，列入省新旧动能转换重大工程项目库个数全省领先，新动能加速成长。

　　时不我待，失之不再。机遇不常有，也不长有。随着重大城市基础设施逐步完善，城市建设将转向内涵式发展，国家支持棚户区改造优惠政策到了最后的窗口期，投入重大基础设施力度将逐步放缓。重大交通设施的完善在吸引要素聚集我市的同时，也有可能引发“倒虹吸”现象。随着各地互联网理念的逐步深入，电子商务从“领先发展”到“技高一筹”需要付出更加艰辛的努力。我市脱贫攻坚由全面推进转向重点区域重点人群，到了攻坚拔寨、啃硬骨头的关键时期，工作难度进一步加大。我们一定增强抢抓机遇的紧迫感、责任感，按照建设现代化经济体系的要求，坚持以创新为第一动力，以协调为内生特点，以绿色为普遍形态，以开放为必由之路，以共享为根本目的，不忘初心、牢记使命，不负重托、奋力拼搏，撸起袖子加油干，付出更多的心血和汗水，在决胜全面小康、实现后来居上的征程中负重奋进。

　　2018年的主要工作预期目标为：地区生产总值增长8.5%左右，规模以上固定资产投资增长10%左右，其中技改投资增长10%，规模以上工业增加值增长9%左右。一般公共预算收入同口径实现增长7%左右。全社会研发投入占地区生产总值比重达到1.45%左右，高新技术产业产值占比达到34.7%。服务业增加值增长10.2%，占地区生产总值的比重提高1个百分点，现代服务业增加值占服务业增加值比重达到52.5%。外贸进出口保持稳定增长。社会消费品零售总额增长10.5%左右，城乡居民人均可支配收入分别增长8.5%和9%左右，居民消费价格涨幅控制在3%以内。PM2.5、PM10等各项指标持续改善，优良天数比例持续增加。围绕实现上述目标，重点抓好以下九个方面。

　　（一）全力推进新旧动能转换，不断提升发展质量效益。坚持质量第一、效益优先，加快建设实体经济、科技创新、现代金融、人力资源协同发展的现代化产业体系，不断增强经济创新力和竞争力。一是突出抓好新旧动能转换重大项目。组建新旧动能转换发展基金，引导社会资本向关键行业和重点领域投放。加快推进石墨烯、新能源电池、北斗导航、航空制造、智能机器人、3D打印等新技术应用项目建设，充分发挥示范引领作用。聚焦“四新” “四化”，突出新一代信息技术、新能源新材料、高端装备制造、冷链物流、医养健康等重点产业，精心策划论证项目200个以上。积极做好项目筛选、申报等工作，争取进入省新旧动能转换重大项目库项目个数位居全省前列。二是深入实施创新驱动发展战略。坚持将创新作为引领发展的第一动力，加快建立企业为主体、市场为导向、产学研深度融合的技术创新体系。着力推进重大创新平台建设，新增省级以上各类创新平台10家，新增省级以上高新技术企业20家，规模以上工业企业设立研发机构的占比超过10%。全面落实人才政策，争取入选泰山产业领军人才5人以上。突出关键共性技术、前沿引领技术、现代工程技术、颠覆性技术创新，争取承担实施省级以上科技计划项目30个，取得重要科技成果100项。启动高新技术产业“铸链”工程，促进科技成果加速转化，着力打造5个产业技术创新战略联盟，力争全市高新技术产业产值突破3000亿元。三是全力推动工业强市战略。坚持增量崛起与存量变革并举，着力改造提升传统优势产业，积极培育特色新兴产业，夯实工业基础。统筹产业、园区和企业发展，支持重点行业、重点产品和关键环节进行数字化、网络化、智能化改造，促进产业向价值链高端迈进，力争全市规模以上工业企业主营业务收入增长8%左右。集中实施110个重大技改项目，重点培育10家循环经济、1家智能制造示范企业，力争全市技改投资突破440亿元。全面实施小微企业治理结构和产业结构“双升”战略，深入开展企业梯次培植计划，努力打造一批细分行业“单项冠军”，新增“四上”企业300家，完成企业规范化改制400家，3家以上企业挂牌上市。大力推进品牌建设，培育地理标志商标6件、中国驰名商标2件，注册马德里国际商标30件，进一步提升产业发展影响力和竞争力。四是创新发展经济新模式新业态。大力实施“归雁兴菏”计划，新建90家返乡创业服务站、1家海外招才引智工作站，探索创建“返乡创业银行”，着力吸引人才、技术、资金等要素回归，推动“打工经济”向“创业经济”转变。积极推进郓城、牡丹区、曹县3个国家级返乡创业试点县建设。努力打造10个市级返乡创业园。新增3.5万名以上在外人员返乡创业，创办领办企业1万家。深入推进国家电子商务示范城市创建工作，持续推动电子商务加快发展，全市淘宝村、淘宝镇分别达到200个、30个以上，电子商务交易额增长50%以上。由“领先一步”转向“技高一筹”，复制推广牡丹创意家居小镇模式，推进互联网与实体经济深度融合，激发跨境电商、定制加工、智慧物流等更多互联网经济新业态。加快“智慧菏泽”、大数据和云计算中心、天华智慧经济产业园、鲁西南大数据产业城等项目建设，建立“云服务劵”财政补贴制度，鼓励更多企业上云、增加用云量，全力推动数字经济发展。打造全国一流的“1688菏泽产业带”。大力发展现代金融业，规划建设菏泽金融聚集核心功能区，力争引进金融机构2家以上。规范发展互联网金融。 继续推进商贸物流和冷链物流标准化企业试点工作，推动商贸物流业提档升级。专项支持发展快递业。大力培育100家重点服务业企业，带动生产性服务业专业化、生活性服务业精细化。五是着力发展园区经济。坚持产业集群化、园区化，由带状、星状经济向块状经济转变，推动产业集约高效发展。支持市经济开发区、菏泽高新区加快转型升级，提升发展层次和水平。加强规划引领，强化基础设施和功能配套，不断健全管理运行机制，大力推进传统工业园区规范化改造，倾力打造县区经济集聚区、菏泽产业融合发展示范园等20个重点经济发展功能区。按照特色小镇建设理念，持续推进菏泽航空制造小镇、牡丹小镇、郓城水浒旅游小镇、单县长寿食品小镇等30个特色产业发展示范区。以文化旅游、健康养老、金融服务等为重点，支持各县区至少打造1处规范化现代服务业集聚区。

　　（二）千方百计扩大有效投资，着力增强发展内生动力。把优化投资结构、提高投资效益放在更加突出的位置，持续实施“三年滚动投资计划”，力争完成固定资产投资1450亿元。一是全力抓好招商引资。持续推动精准招商，加强招商专业人才队伍建设，完善招商政策和考核办法，落实“飞地经济”政策，汇聚招商引资强大合力。灵活运用园区招商、产业链招商、以商招商、委托招商等方式，提高招商实效。加大“腾笼换鸟”力度，突出盘活低效闲置资产，在存量招商上实现重大突破。瞄准重点区域、重点企业、重点行业，深化与各地商会协会等组织合作，有针对性地组织开展系列精准化、专业化招商活动。二是持续推进项目建设。强化签约项目跟踪督导，建立健全重点项目调度和推进机制，加快项目建设进度。突出抓好省市重点项目建设，确保完成年度投资计划。切实抓好市级重大项目库动态管理，形成“投产一批、开工一批、储备一批、谋划一批”的项目梯次推进格局。三是积极拓展融资渠道。用足用好各类产业基金、城投基金和政策性资金，最大限度争取棚改、易地扶贫搬迁等融资额度。深入拓展投资空间，严格落实相关支持政策，鼓励和吸引各类资本进入。充分发挥我市6家投融资公司平台作用，加大资产注入力度，提升运营水平，以市场化手段募集更多社会资本。稳步推进政府和社会资本合作，突出项目策划、推介和集中招商，提高项目规范化水平，加快落地实施步伐。四是加大金融支持实体经济力度。强化存贷比考核，引导金融机构不断扩大信贷投放、回归本源，服务实体经济发展。强化政银企对接。创新中小微企业信贷方式，有效缓解企业资金难题。持续加强金融监管，严厉打击非法集资、恶意逃废银行债务行为，防范化解企业资金链、担保链风险，确保守住不发生系统性、区域性金融风险的底线。

　　（三）实施乡村振兴战略，推动农业农村现代化。按照“产业兴旺、生态宜居、乡风文明、治理有效、生活富裕”的总体要求，深化农业供给侧结构性改革，不断完善公共服务体系，推动农业发展、农村繁荣、农民增收。一是提升农业产业水平。加快发展规模农业。推进农村土地“三权分置”，健全体制机制，完善流转平台，新增土地流转面积20万亩、流转率达到26％。加快发展园区农业。实施“三园同建”，促进“三链重构”、三产融合，抓好 “新六产”示范创建，高标准规划建设各类现代农业产业园区20个。加快发展加工农业。推进农产品精深加工和特色加工，新增农业龙头企业15家，农产品加工转化率提高2个百分点。积极创建木业精深加工示范区。加快发展品牌农业。推进标准化生产，加强质量安全监管，新增省级知名农业品牌3个、区域公用品牌1个、农产品质量安全县3个、“三品一标”产品100个以上。加快发展牡丹产业。突出抓好龙头企业培育、新产品研发和品牌打造，推进出口牡丹加工区建设。大力培育新型农业经营主体。新增市级以上 “一村一品”示范镇20个、示范家庭农场10家，培育“新农人”100名以上。加快发展“互联网+农业”。推动互联网技术在农业生产、监管、营销、服务等环节的充分应用，农产品电商交易额增长50%以上。创新丰富中国林交会办会方式，赋予更多农博会内涵。二是打造生态宜居美好家园。深入推进城乡建设用地增减挂钩和宅基地政策改革，探索实施“地票制”交易，整合各种扶持资金，按照“小组微生”模式，新建一批5000人左右的新型农村社区，复垦增加耕地5万亩以上，打造一批美丽乡村新样本。开展农村环境综合整治，实施垃圾、厕所、污水综合治理。推进村庄绿化亮化美化，打造一批美丽村落、美丽庭院、美丽田园。实施乡村记忆工程，加强非物质文化遗产保护、修复和传承，打造一批集乡土建筑和乡村民俗为一体的综合性、活态化乡村博物馆。实施农村公路提升工程，推进路域治理，加快“四好农村公路”建设，将农村公路打造成靓丽的乡村风景线。三是推进乡村治理现代化。建立健全自治、法治和德治相结合的乡村治理体系。积极打造智慧乡村，提升 “为村”运行管理水平，打造全方位服务村民的平台。积极推进乡村“微自治”，深入实施村居协商制度。积极选树新乡贤，大力培育乡贤文化。开展“菏泽孝贤”命名表彰活动。四是促进城乡深度融合发展。推动城乡产业融合，发展城郊型农业，推进“农社” “农超” “农校”对接，完善农产品配送服务，提高农业效益。推动城乡基础设施融合，充分利用上级支农政策，完善农村供电、供水、通讯、物流、信息等基础设施建设，加快公交、环卫、供气、水电一体化。推进城乡公共服务融合，加快推进教育联合体、医疗联合体、医养联合体、文娱联合体建设，促进城乡居民享受均等社会服务。

　　（四）加快区域中心城市建设，打造宜业宜居城市。坚持完善城市功能配套，不断提升城市发展内涵。一是实施20项重大工程。2020年我们将迎来建市20周年。以此为契机，从今年开始集中实施一批市政公益类标志性工程项目，主要是菏泽机场、京九高铁菏泽段、鲁南高铁菏泽段、市民文化中心、会展中心、市政务服务中心、博物馆、档案馆、市立医院新院区、山东科学院菏泽分院、市职教园区、体育公园、广播影视中心、环堤公园改造提升、中华路改造、人民路北延连接国花大道、人民路与南外环互通立交桥等。二是完善城市交通框架体系。实施城市畅通工程。加快德上高速巨野至单县段、枣菏高速菏泽段、阳新高速菏泽段、日兰高速巨野至菏泽段扩建工程建设。加快新万福河、郓城新河等航道和巨野、定陶等4大港区建设。做好市区主干道与机场、高铁、高速公路的对接，开工建设大学路郭楼东路至水库西二路段、中山路牡丹路至点将台路段等7条道路贯通工程，改造牡丹路黄河路至长江路段、北外环路人民路至广州路段、南京路长江路至闽江路段等15条道路，新建改造国省道一级公路280公里。实施市区断头路攻坚行动和市区路口渠化便利工程，畅通城市微循环。三是增强城市服务功能。强化规划引领作用，完成《菏泽市城市总体规划》和城市总体设计，老城区、高铁新区、双河立交桥片区等城市设计，做好“规划一张图”平台建设和“城市双修”规划的编制。完善城市和小城镇建设体系，加快推进重点镇建设，深化户籍制度改革，推动农村人口加速向社区、乡镇驻地、县城、市区转移，年内实现“三个市民化”16万人。加快推进棚改步伐，全市完成81个片区、12.7万套的棚改拆迁任务。实施市区管网建设攻坚行动，铺设燃气管网15公里、供热管网12公里、综合管廊8公里，建成海绵城市10平方公里。四是提升城市管理水平。深化城市管理执法体制改革，探索运用大数据、云计算加快推进智慧城管、智慧环卫建设。实施中心花园、体育公园、环堤公园、城市绿道及新建市区道路绿化美化，形成网络化的城市绿地系统。大力发展绿色建筑和装配式建筑。加大“两违”建设打击力度。盘活低效闲置土地1万亩。利用平台公司推动土地一级开发，收储土地1万亩，提高经营城市水平，建立发展长效机制。开展城区市容环境综合整治、交通秩序集中整治和物业管理服务规范年活动，努力为广大市民创造良好的生活环境。

　　（五）深化改革扩大开放，切实增强发展活力。大力推进改革创新，以更高层次扩大开放，进一步激发增长动力和发展活力。一是深化供给侧结构性改革。坚持“破、立、降”并举，进一步推动更多优质市场要素向高质量发展聚集。大力破除无效供给，加快处置“僵尸企业”，推动化解过剩产能。深入开展“四评级一评价”行动，有序推动化工行业转型升级。全面落实国家和省降低实体经济成本政策，进一步清理涉企收费，降低企业负担和成本。加快住房制度改革和长效机制建设，保持房地产市场平稳健康发展。二是深化经济领域改革。积极搞好农村集体产权制度改革，完成清产核资和成员资格认定80%以上。深化预算管理改革，完善全口径预算编制，加快乡镇国库集中支付改革。深入落实支持非公有制经济发展的各项改革措施，激发发展活力。加强国资监管，推进国企改革发展。加快推进菏泽国家级普惠金融改革试验区建设，着力构建普惠金融体系。三是深化政务服务改革。深入推进“放管服”改革，以“最多跑一次”改革为目标，推出事项清单，健全“一门办理、并联审批”机制，全面开展容缺受理审批服务。推行“互联网+政务服务”，基本完成全市政务信息系统整合共享，实行网上办事、网上监管，形成线上线下相结合的政务服务新模式，让数据多跑路、百姓少跑腿。四是优化对外开放格局。积极融入“一带一路”建设。推动外贸优进优出，促进外贸提质增效。重点培育机电、户外家具、牡丹产品等新兴出口产业，鼓励先进设备和关键技术进口。积极打造对外平台，加快推进临港产业园规划建设，做好保税物流中心B型申报工作，不断完善青岛保税港区菏泽内陆港功能，确保济铁物流园海关监管场站通过验收。支持跨境电商、外贸综合服务等外贸新业态发展。

　　（六）深入推进精准扶贫，坚决打赢脱贫攻坚战。按照精准扶贫、精准脱贫方略，适应新阶段脱贫实际，狠抓“八个落实”，年内完成脱贫7.2 万人。一是聚力重点群体重点区域。实施深度贫困群众兜底脱贫，建立低保、养老、教育、医疗“四位一体”的社会保障机制，进一步提升脱贫质量。推动黄河滩区脱贫迁建工程，加快村台和外迁社区建设，建成村台18个。落实滩区产业发展规划，完善配套基础设施，改善滩区群众生产生活条件。二是深化“三个一”扶贫工程。深化“一户一案”精准扶贫，注重扶贫同扶志、扶智相结合，激发贫困群众内生动力。完善“双向承诺”机制，逐户逐人细化帮扶方案。深化“一村一品”产业扶贫，围绕贫困人口脱贫需求，有选择、有重点地发展扶贫产业。深化“一人一岗”就业扶贫，提升扶贫车间建设、运营、管理水平，扩大贫困群众用工，让贫困户稳定获得收入。三是更加注重解决“三保障”。落实建档立卡贫困户学生资助制度，坚持长效常态公开管理，使每名贫困家庭学生都能享受到教育扶贫政策。及时落实危房改造补助资金，集中解决 “四类重点对象”的基本住房安全问题。落实医疗帮扶政策，全面推进贫困家庭基本医疗保险、大病医疗保险、大病补充医疗保险全覆盖，加大医疗救助、临时救助、慈善救助，进一步减轻贫困群众医疗负担，防范因病返贫致贫。四是持续加大扶贫资金投入。健全资产收益扶贫机制，推动资源变资产、资金变股金、农民变股东。打好财政、银行、保险“组合拳”，建立扶贫基金，形成多元化、多层次、多渠道的扶贫投融资体系。加大涉农资金整合力度，新增涉农资金集中用于脱贫攻坚。严格执行脱贫攻坚问责办法，从严查处侵害贫困群众利益的突出问题，确保脱贫工作经得起人民、实践和历史的检验。

　　（七）全面强化环境保护，打造生态宜居菏泽。牢固树立绿色发展理念，坚决落实环保督察整改任务，标本兼治推进环保工作，不断满足人民日益增长的优美生态环境需要。一是坚持铁腕治污。持续推进“气十条”，突出降尘、减煤、治气、控车等重点，重拳治理大气污染，打好蓝天保卫战，确保PM2.5、PM10等各项大气指标持续改善。提高整治标准，持续推进“散乱污”企业治理。深入实施“水十条”，突出污水收集处理、河道综合整治、工业废水外排标准提升等重点，加大水污染治理，确保三条主要出境河流断面水质稳定达标。全面实施“土十条”，推进土壤污染监管、治理和修复，加大农村面源污染整治，持续改善永久基本农田土壤环境质量。加大环保执法力度，严惩环境违法行为，巩固环保治理成果。二是推动绿色发展。大力倡导绿色生活、绿色消费、绿色出行，让绿色理念深入人心。严格落实能源和水资源消耗、建设用地等总量和强度“双控”，提高节能、节水、节地、节材标准。加快发展节能环保产业，提升环保技术装备和产品供给能力。深入推进绿色菏泽建设行动，加快生态修复，打造优美生态，争创国家卫生城市、森林城市和生态园林城市。三是构建长效机制。深化生态文明体制改革，全面推行环保机构监测监察执法垂直管理制度。加强环保网格化监管体系、智慧化监管平台建设。严格落实“河长制”，实现全市河段生态保护与治理责任的全覆盖。健全生态补偿机制，探索建立生态环境损害赔偿制度。完善环保行政执法与刑事司法衔接联动机制，强化环保督察，加强环保治理目标责任考核，严格执行生态环境损害责任终身追究制，推动生态文明建设走向制度化、常态化。

　　（八）加强和创新社会治理，完善现代治理体系。坚持共建共治共享，推进社会化、法治化、智能化、专业化治理有机融合，不断提升社会治理科学化水平。一是深化平安菏泽建设。推进立体化社会治安防控体系建设，建立覆盖全市的公共安全视频监控网络。深入开展打霸治痞、扫黑除恶专项行动。深入推进道路交通秩序专项治理。健全公共安全体系，强化安全生产风险分级管控和隐患排查治理责任双重预防体系，深入开展隐患综合排查行动，坚决遏制较大以上生产安全事故。积极开展省级食品安全城市和食品安全先进县区创建活动，打造食品药品安全示范区。发展气象、水文、地质、地震等事业，增强综合防灾减灾救灾能力。二是提升法治菏泽水平。健全均等普惠型公共法律服务体系，全面实施一村一法律顾问。加大法律法规宣传和咨询服务力度，完善矛盾纠纷多元调解机制，促进群众合理诉求依法及时解决。加快社会信用体系平台建设，整合社会资源褒扬诚信、惩戒失信。依法管理民族宗教和外事侨务等工作。三是推动社会治理重心下移。深化社区管理体制改革，推进政社互动、社区减负，逐步完善基层社会治理体系。推动治理重心向村居、社区转移，向社会需求、群众诉求转移，促进政府治理与社会自我调节、居民自治良性互动。健全政府购买服务常态机制，发展公益性、服务性、互助性社会组织。培育发展各类慈善主体，激发慈善工作活力。加强后备力量建设，提高国防动员能力。巩固双拥创建成果，促进军民融合深度发展。

　　（九）强化公共服务能力，持续增强民生福祉。围绕补齐补强民生短板，不断完善公共服务体系，切实增强人民群众的获得感、幸福感。一是优先发展教育事业。强化优质教育资源辐射能力，推动校长职级制、中小学教师“县管校聘”和义务教育学校校长教师交流改革，改扩建义务教育学校100所以上、补充教师4000名以上。健全中小学办学行为动态监管机制，严格规范办学行为，鼓励社会力量办学，推动义务教育由“基本均衡”向“优质均衡”迈进。加强学前教育。大力发展职业教育，建成市职教园区，推动职业教育产教融合、校企合作。二是提高社会保障水平。优化就业环境，完善就业平台，扩大就业培训，推动重点群体就业，新增城镇就业6.5万人。全面实施全民参保计划，稳妥推进机关事业单位养老保险制度改革，推动基本医保全国联网和异地就医结算。加强困境儿童生活保障，建立健全基层关爱阵地和服务网络，提升农村“三留守”关爱保护水平。推动医养结合向纵深发展，全市新增养老床位3500张。三是推进健康菏泽建设。深化公立医院综合改革，完善分级诊疗制度，加快医联体建设，推动优质医疗资源下沉。推进二、三级医院标准化建设，实施县级医院服务能力提升工程，县域内就诊率达到90%左右，基层医疗卫生服务体系实现城乡全覆盖。推动中医药事业创新发展。深化生育全程服务，加强出生缺陷综合防治，优化人口性别比结构。提高婴幼儿照护和儿童早期教育水平。推广全民健身活动联赛，举办各类比赛2000场次以上。四是推动文化繁荣发展。弘扬优秀传统文化、红色文化。加强文化遗产保护，新建非遗特色社区3处、非遗传习场所20处。实施文化惠民工程，开展冬春文化惠民季、农村文化艺术节、送戏下乡等群众文化活动，支持优秀民营艺术团体健康发展。统筹整合全市A级景区旅游资源，打造精品线路，创新营销宣传模式，推动全域旅游加快发展。五是抓好10个方面的民生实事。（1）打通市区全部42条“断头路”；（2）完成市区主要道路52个路口的全部渠化改造；（3）新建改造市区污水管网138公里，实现污水管网全覆盖；（4）在市区规划建设100处以小绿地、小公园、小广场和小停车场为主题的“口袋公园”；（5）在市区牡丹路、黄河路各建一座人行天桥；（6）开通市区到定陶及各县的城际公交；（7）新建改造市区公共厕所20座、农村厕所43万户；（8）对5.7万对夫妇开展免费孕前优生健康检查，实施农村适龄妇女 “两癌”免费检查8.9万人，实施1万名残疾儿童康复工程，实施重性精神疾病患者医疗救治救助项目；（9）新建城市社区老年人日间照料中心20处、农村幸福院266处，推进56处中心敬老院规范化管理；（10）新建农村公路2000公里，实现自然村“村村通”，启动“户户通”工程。

　　三、全面加强政府自身建设

　　新时代、新使命，对政府自身建设提出了新的更高要求。要把牢政治方向，加快转变职能，强化责任担当，持续打造高素质专业化的干部队伍，努力建设人民满意政府。

　　一是坚持忠诚担当。牢固树立政治意识、大局意识、核心意识、看齐意识，坚定不移维护习近平总书记的核心地位、领袖权威。坚决贯彻落实党中央国务院、省委省政府和市委各项决策部署。把对党忠诚、为党分忧、为党尽职、为民造福作为根本政治担当，扎实开展“不忘初心、牢记使命”主题教育，始终把人民对美好生活的向往作为奋斗目标，把群众路线贯穿政府工作各领域全过程，努力担负起政府推进改革发展的重任。

　　二是坚持提升本领。深入学习贯彻习近平新时代中国特色社会主义思想，用党的创新理论武装头脑、推动工作。为政之要在于勤学习、善研究、重实干。要着力提升学习本领、政治领导本领、改革创新本领、科学发展本领、依法执政本领、群众工作本领、狠抓落实本领、驾驭风险本领，锤炼定力深、眼力准、能力强、合力好的干部，以更高的素质、更高的能力适应新形势、推动新发展。健全完善干部激励评价机制，关心关爱基层干部，营造敢于担当、踏实做事、不谋私利、积极向上的成长环境，进一步促进各级干部锐意进取，干事创业，不辱使命。

　　三是坚持依法行政。严格遵守宪法和法律，善于用法治思维和法治方式加快改革、推动发展、化解矛盾，把政府工作全面纳入法治轨道。严格执行重大行政决策、规范性文件制定法定程序，积极发挥政府法律顾问作用，推进政府“决策、执行、管理、服务、结果”全公开。全面推行行政执法公示制度、执法全程留痕制度、重大执法决定法制审核制度，全面规范行政执法行为。自觉接受市人大及其常委会的法律监督、工作监督和市政协的民主监督，健全与人大代表、政协委员的沟通联络机制，高质量办好建议提案，认真听取各民主党派、工商联、无党派人士和社会各界意见。全力支持国家监察体制改革试点工作，自觉接受监察委员会的监督。深入推进政务公开，充分发挥新兴媒体作用，利用好网络平台，及时回应社会关切，主动接受社会监督，让权力公开透明、阳光运行。

　　四是坚持廉洁勤政。认真落实党风廉政建设主体责任和“一岗双责”，深入推进廉政建设。把纪律和规矩挺在前面，运用好监督执纪“四种形态”，促进各级干部知敬畏、存戒惧、守底线。持续加强作风建设，驰而不息纠正“四风”，深入落实中央八项规定实施细则精神和省委实施办法，始终保持清正廉洁本色。加强审计监督，强化对环境保护、工程招投标、房屋征收、脱贫项目等重点领域监管，持续深入查处各种不正之风和发生在群众身边的腐败行为。以科学的方法狠抓工作落实，建立健全责任明晰的重点工作清单机制、严格高效的考核激励机制、常态长效的追责问责机制，以永不懈怠的精神状态扎实做好各项工作。

　　各位代表！新时代孕育新希望，新思想引领新征程。新的历史方位，赋予我们新的历史担当。让我们更加紧密地团结在以习近平同志为核心的党中央周围，深入贯彻习近平新时代中国特色社会主义思想，在中共菏泽市委的坚强领导下，团结依靠全市人民，解放思想，凝心聚力，开拓创新，埋头苦干，持续发力，久久为功，为决胜全面小康、实现后来居上的宏伟目标而努力奋斗！
